# Inpatient management of borderline personality disorder at Helen Joseph Hospital, Johannesburg

**DOI:** 10.4102/sajpsychiatry.v22i1.678

**Published:** 2016-06-17

**Authors:** Laila Paruk, Albert B.R. Janse van Rensburg

**Affiliations:** 1 Department of Psychiatry, University of the Witwatersrand, South Africa; 2 Helen Joseph Hospital, South Africa

## Abstract

**Objective:**

The aim of this report was to establish a profile of patients with borderline personality disorder (BPD) admitted to the acute inpatient psychiatric assessment unit at the Helen Joseph Hospital, in Johannesburg, over the course of 1 year.

**Methods:**

A retrospective record review was conducted to investigate the prevalence, demographics, reasons for admission, treatment, length of stay and follow-up of a group of inpatients during 2010 with a diagnosis of BPD, based on DSM-IV-TR diagnostic criteria, allocated on discharge.

**Results:**

Considering evidence retrospectively, the quality of the BPD diagnosis allocated appeared adequate. Statistical analysis revealed findings mainly in keeping with other reports, for example, that patients with BPD are above-average users of resources who make significantly more use of emergency services and that they generally do not adhere well to their scheduled outpatient follow-up arrangements. The longer average length of inpatient stay of this group with BPD, however, exceeded the typically brief period generally recommended for acute inpatient containment and emergency intervention.

**Conclusion:**

Implementation of targeted prevention and early intervention strategies, based on systematised programmes such as dialectical behavioural therapy and mentalisation based therapy, may be useful in addressing these problems experienced with integrating the in- and outpatient management of BPD.

## Introduction

According to Davison:

… the management of patients with personality disorder is one of the most challenging and sometimes controversial areas of psychiatry.^1^

They have many diverse needs, and often present repeatedly to psychiatric services. The *Diagnostic and Statistical Manual of Mental Disorders* (DSM IV-TR Edition) clustered the 10 identified personality orders in three groups: Cluster A, B and C, with Cluster B including histrionic, narcissistic, borderline and antisocial personality disorders. The DSM IV-TR characterises borderline personality disorder (BPD) as:

A pervasive pattern of instability of interpersonal relationships, self-image and affects, and marked impulsivity beginning by early adulthood and present in a variety of contexts.^2^

It describes nine criteria, of which five must be fulfilled in order for a diagnosis of BPD to be made. A fifth edition of the DSM (DSM 5) was introduced in May 20133; however, there have been no significant changes to the description of personality disorders (Appendix 1).

A person is considered to have borderline personality traits if exhibiting less than five symptoms of BPD. The determining principle is the DSM IV-TR ‘Criterion C’ for a personality disorder, that:

… the (deviating) enduring pattern (of inner experience and behaviour) must lead to clinically significant distress or impairment in social, occupational, or other important areas of functioning.^2^

Recent research into the epidemiology of borderline personality has shown that it affects 0.7% – 2.7% of the general adult population, 9.3% – 22.5% of people receiving psychiatric outpatient treatment, and in some settings over 40.0% of inpatients.^4^ BPD is frequently co-occurring with affective disorders, anxiety disorders, somatisation disorder, post-traumatic stress disorder and alcohol abuse, while a differential diagnosis of bipolar disorder (BD) often has to be considered. Patients with BD present more often with emotional lability, whereas BPD patients are characterised by intense and reactive affective instability and shifts from sadness to tolerable dysphoria.^5^

Consequently, patients with personality disorders make frequent use of health services, in particular emergency services.^6^ Crises related to depression and suicide account for approximately 30.0% of the cases that present to psychiatric emergency services.^7^ According to Links, these suicide threats and attempts are defining criteria for the disorder.^6^ Another study by Dowson and Grounds showed that patients with personality disorders have higher rates of suicide and accidental deaths than the general population.^8^

Although Fagin considers acute inpatient units generally to be unsuitable for long-term work with people with personality disorders,^9^ Norton and Hinshelwood believe that ‘an admission …, although often problematic, can be conceived as an opportunity’.^10^ Inpatient admission to a general psychiatric ward, however, should usually be brief, time-limited and goal determined, and a patient may be discharged if the goals of admission are not met, according to Bateman and Tyrer.^11^

Helen Joseph Hospital (HJH) is a regional specialist referral state hospital in Auckland Park, Johannesburg, and a teaching facility affiliated with the University of the Witwatersrand (WITS). The psychiatric unit (Ward 2) at HJH is a 30-bed acute unit for adult users and is designated to provide 72-h assessment as well as emergency and short term inpatient psychiatric care. The unit aims to provide a therapeutic milieu in which patients with BPD may be managed. On admission, patients contract not to resort to aggression, self-harm, substance use and not to develop intimate relationships on the ward.

On discharge, patients are directed to follow up either at their local community clinic, or at the HJH Psychiatry and Psychology outpatients. Following discharge, the Department of Psychology at HJH offers outpatient groups to assist patients with life skills and individual therapy. These groups are based on the principles of dialectical behavioural therapy (DBT) as well as mentalisation based therapy (MBT).

There is also the option within the WITS group of referral facilities for patients to be referred for a 5–6 week inpatient programme to the psychotherapy unit (Wards 4 and 5) at Tara the H. Moross Centre (Tara Hospital), which is a public-specialised psychiatric facility in the north of Johannesburg.

Previous data describing the clinical profile of mental healthcare users at HJH showed that the average number of admissions per year over the 5 years from 2004 to 2008 was 535, and the average length of stay was 15.4 days.^12,13^ Twenty-four per cent of these patients admitted in 2003 and 2004 had a diagnosis of cluster B personality traits or disorder, while in 2007 and 2008 this figure was 27.3% (*n* = 119). These figures provided preliminary information regarding the number of inpatients with BPD at HJH. However, little other data are available on patients with BPD in South Africa.

The purpose of this explorative study was therefore to review the frequency, management and outcome of the acute inpatient treatment of patients with BPD at HJH. The objectives of this study were to:

- Establish the percentage of inpatients with BPD.
- Describe the demographic and clinical profile of these patients with BPD.
- Review the number of these patients’ psychiatric outpatient and emergency or consultation-liaison visits.

## Methods

The study was a retrospective, descriptive, clinical review of all the inpatients with BPD at the acute adult psychiatric assessment unit (Ward 2) at HJH over 1 year. Ethics clearance was obtained from the Human Research Ethics Committee of the University of the Witwatersrand.

### Data collection

Data were sourced from patients’ clinical records and an existing electronic database of admissions to the unit. Admission records, clinical notes and discharge summaries were reviewed.

### Data analysis

Firstly, to assess the quality of the diagnoses of BPD documented for these inpatients, the patients who have been allocated a diagnosis of BPD or borderline traits by doctors on discharge and those for whom evidence was retrospectively found in the clinical file to actually fulfil the diagnostic criteria were compared using a chi-square test to determine significance, which was set at *p* = 0.05. Other variables were then described, presenting categorical variables as frequencies and percentages, and continuous variables as a mean with standard deviations (SD) if normally distributed, or as a median (range) if not normally distributed.

## Results

The total number of patients admitted to Ward 2 during 2010 was 653. Of this number, the total identified from the ward’s admission records as diagnosed with ‘BPD’ or with ‘borderline personality traits’ was 121 (18.5% of the total). The clinical records of 24 patients were not available (*n* = 24; 3.6%); the sample therefore included 97 patients, 14.8% of the total (*n* = 653).

### Confirmation of diagnosis

Patients were divided into two sets of two groups each (Table 1). The first set (*n* = 97) was those who were documented by the treating doctor on discharge to have a diagnosis of BPD (*n* = 75), or those who only had borderline personality traits (*n* = 22). The second set was identified, after critically reviewing the clinical file data for evidence, as those who actually fulfilled all the DSM IV-TR criteria for BPD (*n* = 45), and those who only had borderline traits (*n* = 15). When comparing these two groups, no statistical significant difference was observed (*p* = 0.14), suggesting that the quality of diagnoses made in the unit was adequate.

**TABLE 1:** Comparing borderline personality disorder diagnoses made from clinical data with DSM IV-TR diagnostic criteria of Helen Joseph Hospital psychiatric inpatients, 2010.

Disorder or traits	Diagnosis per clinical records on discharge	Diagnosis per DSM IV-TR diagnostic criteria
Borderline personality disorder	75	45
Borderline personality traits	22	15

*p* = 0.14

### Demographic profile

The demographic data of patients with BPD admitted to HJH in 2010 are summarised in Table 2, demonstrating that the majority were younger, white females.

**TABLE 2:** Demographic information.

Variable	Percentage
**Age in years**	
18 – 29	31
30 – 39	31
40 – 49	28
> 50	7
Unknown	4
**Gender**	
Female	79.8
Male	21.2
**Race**	
Asian	3.1
Black people	15.4
Mixed Race	9.3
White people	72.2

#### Clinical profile

**FIGURE 1:**
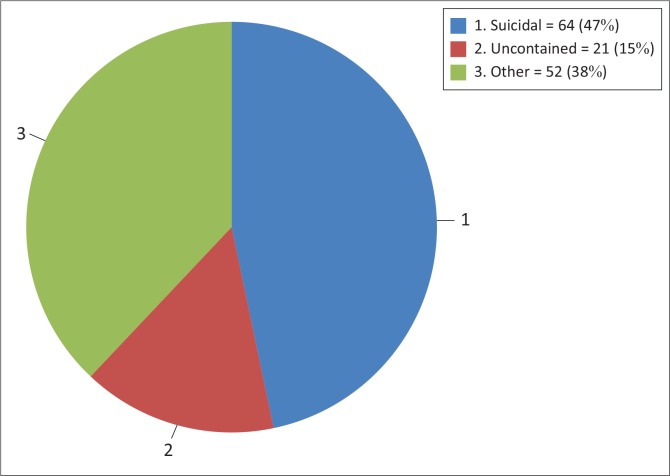
Reasons for admission of patients with borderline personality disorder admitted to the acute psychiatric unit at Helen Joseph Hospital during 2010.

**FIGURE 2:**
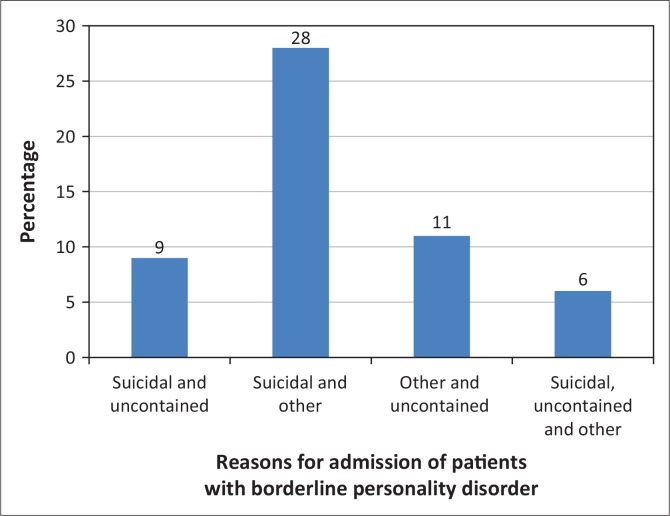
Multiple reasons for admission of patients with borderline personality disorder admitted to the acute psychiatric unit at Helen Joseph Hospital during 2010.

**Referral:** Sixty-four patients presented with suicidal ideation, 21 were uncontained and 52 were admitted for ‘other’ reasons (Figure 1). ‘Other’ included mood lability, substance withdrawal and psychotic episodes. This number being in excess of the sample size (*n* = 137) became apparent that some patients were admitted for more than one reason. The data were further analysed to assess how many patients had multiple reasons for admission, and what the overlap was (Figure 2). Twenty-eight patients had both suicidal ideation and other reasons, while six patients were admitted with the threefold suicidality, ‘uncontained’ and ‘other’.

**Co-morbidity:** While some patients were admitted with only a diagnosis of borderline personality (Table 3), 42 (*n* = 42, 42.0%) had a co-morbid substance problem. BD, including both types 1 and 2, accounted for 15.0% of the co-morbidities documented (*n* = 15, 15.0%).

**TABLE 3:** Co-morbidities of patients with borderline personality disorder admitted to the acute psychiatric unit at Helen Joseph Hospital during 2010.

Diagnosis	Number	%
Substance abuse/dependence	42	43
Bipolar disorder (1 and 2)	15	15
Major depressive disorder	13	13
Substance-induced disorders	4	4
Adjustment disorder	3	3
Eating disorders	3	3
Psychotic disorders	2	2
Post-traumatic stress disorder	1	1
Paraphilia	1	1
Malingering	1	1

**FIGURE 3:**
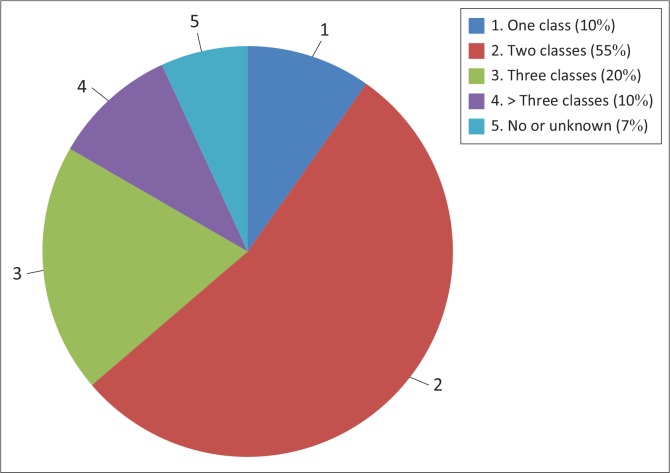
Number of classes of medication on discharge of patients with borderline personality disorder at Helen Joseph Hospital during 2010.

**Treatment:** Data on the use of pharmacological agents were described by *classes* of medication that patients were discharged on (Figure 3). Ten patients were discharged on one class of medication (*n* = 10, 10.3%), 55 were discharged using 2 (*n* = 55, 56.7%), 20 using three (*n* = 20, 20.6%) and 10 were discharged on more than three classes of medication (*n* = 10, 10.3%). Five patients were discharged on no pharmacology (*n* = 5, 5.15%), and the data on two patients were incomplete (*n* = 2, 2.06%).

**FIGURE 4:**
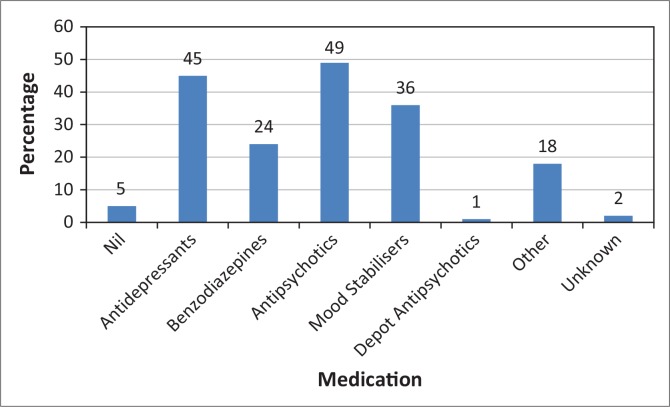
Type of medication on discharge of patients with borderline personality disorder admitted to the acute psychiatric unit at HJH during 2010.

Forty-five patients were being treated with antidepressants (*n* = 45, 46.4%); whereas 24 were using benzodiazepines (*n* = 24, 24.7%). Forty-nine patients were prescribed an oral antipsychotic (n = 49, 50.5%) and one was prescribed a depot antipsychotic (*n* = 1, 1.0%) (Figure 4). Thirty-six patients were discharged on a mood stabiliser and 18 on other medication, including those prescribed for systemic illnesses.

**Length of stay and referral:** The average length of stay for patients with BPD admitted to the acute psychiatric unit at HJH during 2010 was 16.5 days, with a SD of 13.44 days and a median of 6 days.

**FIGURE 5:**
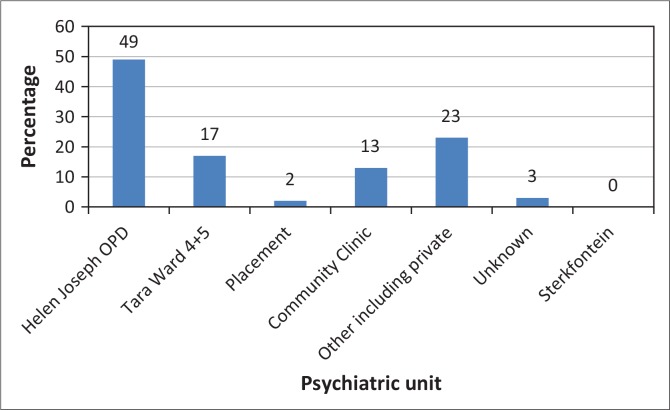
Discharge plans for patients with borderline personality disorder admitted to the acute psychiatric unit at Helen Joseph Hospital during 2010.

On discharge patients were either referred to continue care as outpatients at HJH or were transferred to other facilities. Figure 5 illustrates the referral plan as given to patients on discharge. The majority were directed to follow up at the HJH outpatient department (*n* = 49, 50.0%), 17 were referred to the Tara Hospital psychotherapy programme (*n* = 17, 17.7%) and 13 to a community clinic (*n* = 13, 14.0%). Two patients were placed at a long-term residential facility, whereas 23 were referred to the private sector, or for substance rehabilitation.

**FIGURE 6:**
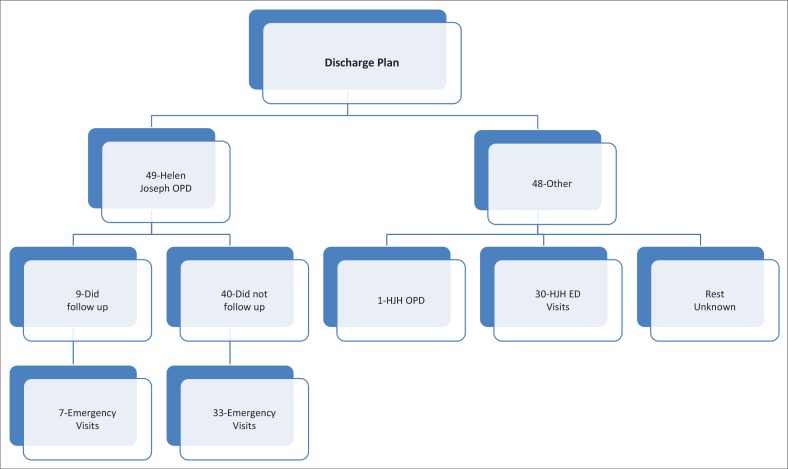
Tracking of patients after being discharged with a diagnosis of borderline personality.

**Outpatient, emergency or consultation-liaison visits:** The actual movements of patients following discharge were compared to the initially proposed plan (par 3.4). Patients were again split into two groups: those who were supposed to follow up at the HJH Psychiatric Outpatient Department (*n* = 49) and those who were supposed to follow up elsewhere (*n* = 48), Figure 6.

The electronic outpatient database for 2010 was then scrutinised to track whether these patients did, in fact, present as scheduled. Of the 49 patients meant to be seen as outpatients at Helen Joseph, only 9 (18.0%) kept their appointments. The data were also cross-referenced against the emergency visits for 2010 while keeping the patients in the same two groups. Seven of the nine patients who were compliant with their outpatient visits also presented as emergency cases during the study period. Thirty-three of the forty (83.0%), who were non-adherent to their outpatient dates, were actually seen as emergency cases.

Of the 48 patients that were given a plan other than following up with HJH outpatients on discharge, 30 (63.0%) presented to the HJH Emergency Department anyway, whereas one returned unscheduled to the HJH Outpatient clinic.

## Discussion

With regard to limitations of the study, Hess noted that:

… retrospective research often requires the analysis of data that was originally collected for reasons other than research.^14^

The limitations of retrospective research thus include incomplete documentation, missing charts, information that is unrecoverable or unrecorded, difficulty interpreting information found in the documents, problematic verification of information and difficulty establishing cause and effect, as well as variance in the quality of information recorded by medical professionals.^15^ The majority of the data for this retrospective review were from clinical and nursing records, which were often incomplete. Twenty-four files, about 20.0% of the sample size, were not found. Of the records that were accessible, discharge summaries were often completed by junior doctors.

Personality disorders are often difficult to pinpoint to a specific clinical (DSM IV-TR) diagnosis, and clinicians may often describe symptoms more broadly within a personality cluster. While the quality of allocated BPD diagnoses considered in this review was regarded to be adequate, the overall results could still be considered to be an *underestimation*, due to the relatively strict inclusion criteria in the study design.

In addition, this report did not incorporate the assessment, criteria and interventions provided by the Department of Psychology at HJH, except to make mention when patients were referred. Ideally it would be useful to follow these patients and compare outcomes with or without psychological intervention. Better information on this process may contribute to a more seamless integrated programme to effect-indicated prevention and early intervention. The study also did not incorporate the follow-up of patients who were referred to the Tara Hospital inpatient psychotherapy programme. This is a voluntary programme, and additional information could have included whether the referred patients presented for their assessment interviews, whether they were accepted in the programme and if they completed the programme. This information could also have been compared with acute admission relapse rates to determine the presence of a relationship between the two.

The total percentage of patients documented with BPD or traits in this study was lower than figures from the international data. These studies, which used research diagnostic instruments, have found that 20.0% – 40.0% of psychiatric outpatients and about 50.0% of psychiatric inpatients fulfil criteria for a personality disorder.^16,17^ The finding at HJH of about 18.5%, therefore, may represent an underestimation, probably because it is based on clinical rather than research criteria.

A large proportion of patients in this study were admitted for more than one reason, which is in keeping with the literature, which reports that people with personality disorders often present in crisis situations and their personality pathology is sometimes secondary and emerges after admission.^9^

The occurrence of polypharmacy with agents from all classes, shown in this study, further illustrates that patients with personality disorders utilise more resources but may also seems to reflect inappropriate prescribing patterns. Especially in view of evidence that pharmacological intervention is not first-line in the treatment of personality disorders and is only useful to target directed symptoms. It may also reflect co-morbidity, as well as the lack of clarity of diagnosis in some instances. The use of habit forming benzodiazepines, in particular, has a limited indication in the management of BPD. Its use in this population with additionally very high rates of co-morbid substance abuse would warrant further attention to prescribing patterns in the HJH inpatient unit.

Comparing this study’s finding of a longer length of stay (16.5 days) for BPD patients to that of the general inpatient population in 2007 (15.4 days),^12,13^ it seems that the objective of the HJH protocol to limit the length of stay of BPD patients had not been achieved during this study period. While acknowledging the usefulness of a short, therapy-intensive admission, a targeted intervention during the acute admission period should include setting a discharge date early to prevent ‘longer-than-necessary’ stays.

As a group, the patients with BPD in this study were largely non-adherent to scheduled follow-up. They presented instead frequently to the HJH Emergency Department for unscheduled emergency psychiatric services. The implications of this include the lack of continuity with named clinicians, and less than optimal after-hours assessments, often by junior staff, resulting in an inefficient use of resources. A targeted programme should at least include an assertive treatment plan which contacts patients who do not present for scheduled visits.

## Recommendations

### Consider all components in the referral system

The study clearly illustrates the burden on emergency versus scheduled care. It may be worthwhile to explore all the service components available to BPD patients in the area. This would include exploring the extent of compliance with the arrangements of the HJH psychology outpatient department, which runs parallel to, but is not integrated with, the discharge recommendation by the HJH psychiatry department.

### Quality of diagnoses

Clinical interviewing using a structured diagnostic tool (or interview) may also yield more accurate results, and so would improve the evidence for a more clear diagnosis of BPD. Future studies may also look into the close relationship between personality disorders, substance use and suicidality as a reason for admission, which emerged from this review.

### Interventions

An acute inpatient facility provides an ideal opportunity for early intervention programmes in BPD. BPD is a leading candidate for developing empirically based prevention and early intervention programmes because it is common in clinical practice, is among the most functionally disabling of all mental disorders, is often associated with help-seeking and has been shown to respond to intervention even in those with established disorder.^18^

The existing programme at HJH may also benefit from incorporating short-stay inpatient and outpatient MBT and DBT principles, as well as additional objectives such as early intervention. Early intervention should primarily aim to alter the life-course trajectory of people with borderline personality pathology by attenuating or averting associated adverse outcomes and promoting more adaptive developmental pathways. Novel early intervention programmes have been developed and researched in Australia and the Netherlands.^19^ These would include elements like:

- Assertive, psychologically informed case management, integrated with the delivery of individual psychotherapy.
- Active engagement of families or carers.
- General psychiatric care by the same team.
- Capacity for outreach care in the community, with flexible timing and location of intervention.
- Crisis team and inpatient care, with a clear model of brief and goal-directed inpatient care.
- Access to a psycho-social recovery programme.
- Individual and group supervision of staff.
- A quality assurance programme.

### Barriers and potential risks

Stigma is still a barrier to the early diagnosis of BPD. It is highly stigmatised among professionals, and it is also associated with patient self-stigma.^20^ Many clinicians deliberately avoid using the diagnosis with the aim of ‘protecting’ the individuals from discriminatory practices.

### Future perspectives

BPD can be seen as a lifetime developmental disorder with ramifications across different life stages. There is now sufficient evidence to support diagnosing and treating the disorder when it first appears in routine clinical practice, that is, in acute inpatient or outpatient settings. This has already been adopted by the NICE guideline and supported by DSM V and likely to be supported by ICD 11.^18^ Prevention and early intervention offers a unique platform for investigating the disorder early in its clinical course, where duration of illness factors that complicate the psychopathology and neurobiology of the disorder can be minimised.

## Conclusion

This review showed that, during the study period, the current protocol in place at HJH did not have its desired outcome in patients with BPD, who were frequently stayed longer, were re-admitted and did not follow up via the appropriate channels. These findings support the development and implementation of a ‘unit-within-a-unit’ structure, where these patients are identified early and embarked upon structured programmes which have a robust basis in literature for improving outcomes, reducing morbidity and thereby preserving resources.

## Appendix 1

Personality disorders, according to criteria of the fourth edition (Text Revision) of the Diagnostic and Statistical Manual of Mental Disorders (DSM IV-TR), are defined as: ‘… an enduring pattern of inner experience and behaviour that deviates markedly from the expectations of the individual’s culture’. This pattern is manifested in two or more of the following areas:

- cognition, that is, ways of perceiving and interpreting self, other people and events;
- affectivity, that is, the range, intensity, lability and appropriateness of emotional responses;
- interpersonal functioning; and
- impulse control

Diagnostic criteria – Borderline Personality Disorder

1. Frantic efforts to avoid real or imagined abandonment
2. A pattern of unstable and intense interpersonal relationships characterised by alternating between extremes of idealisation and devaluation
3. Identity disturbance markedly and persistently unstable self-image or sense of self
4. Impulsivity in at least two areas that are potentially self-damaging (spending, sex, substance abuse, reckless driving, binge-eating)
5. Recurrent suicidal behaviour, gestures or threats, or self-mutilating behaviour
6. Affective instability due to marked reactivity of mood (e.g. intense episodic dysphoria, irritability or anxiety usually lasting a few hours and only rarely more than a few days)
7. Chronic feelings of emptiness
8. Inappropriate, intense anger or difficulty controlling anger (frequent displays of temper, constant anger, recurrent physical fights)
9. Transient, stress-related paranoid ideation or severe dissociative symptoms

The fifth edition of the *Diagnostic and Statistical Manual of Mental Disorders* (DSM V) was recently introduced in May 2013.^1^ During the development of this edition, several proposed revisions were drafted that would have significantly changed the method by which individuals with personality disorders are diagnosed. Based on the feedback from a multilevel review of proposed revisions, the American Psychiatric Association Board of Trustees ultimately decided to retain the DSM IV-TR categorical approach with the same 10 personality disorders. The proposed revisions that were not accepted for the main body of the manual were approved as an alternative hybrid dimensional–categorical model that will be included in a separate chapter of DSM V. This alternative model has been included to encourage further study on how this new methodology could be used to assess personality and diagnose personality disorders in clinical practice. DSM V, however, moved from the previously used multiaxial diagnostic system to a new assessment format that removes the arbitrary boundaries between personality disorders (previously documented on Axis II) and other mental disorders, by collapsing the different dimensional axes into one diagnostic statement.
